# The Complete Chloroplast Genome Sequence of *Eupatorium fortunei*: Genome Organization and Comparison with Related Species

**DOI:** 10.3390/genes14010064

**Published:** 2022-12-25

**Authors:** Kan Yan, Juan Ran, Songming Bao, Yimeng Li, Rehmat Islam, Nai Zhang, Wei Zhao, Yanni Ma, Chao Sun

**Affiliations:** 1 School of Biological and Pharmaceutical Engineering, Lanzhou Jiaotong University, Lanzhou 730030, China; 2 School of Pharmacy, Lanzhou University, Lanzhou 730000, China; 3 Key Laboratory of Space Bioscience and Biotechnology, School of Life Sciences, Northwestern Polytechnical University, Xi’an 710072, China; 4 College of Agronomy, Gansu Agricultural University, Lanzhou 730101, China

**Keywords:** *Eupatorium fortunei*, chloroplast genome, comparative analysis, phylogenetic analysis

## Abstract

*Eupatorium fortunei* Turcz, a perennial herb of the Asteraceae family, is one of the horticultural and medicinal plants used for curing various diseases and is widely distributed in China and other Asian countries. It possesses antibacterial, antimetastatic, antiangiogenic, and antioxidant properties along with anticancer potential. However, the intrageneric classification and phylogenetic relationships within *Eupatorium* have long been controversial due to the lack of high-resolution molecular markers, and the complete chloroplast (cp) genome sequencing has not been reported with new evolutionary insights. In the present study, *E. fortunei* was used as an experimental material, and its genome was sequenced using high-throughput sequencing technology. We assembled the complete cp genome, and a systematic analysis was conducted for *E. fortunei*, acquiring the correspondence of its NCBI accession number (OK545755). The results showed that the cp genome of *E. fortunei* is a typical tetrad structure with a total length of 152,401 bp, and the genome encodes 133 genes. Analysis of the complete cp genomes of 20 Eupatorieae shows that the number of simple sequence repeats (SSRs) ranged from 19 to 36 while the number of long sequence repeats was 50 in all cases. Eleven highly divergent regions were identified and are potentially useful for the DNA barcoding of Eupatorieae. Phylogenetic analysis among 22 species based on protein-coding genes strongly supported that *E. fortunei* is more closely related to *Praxelis clematidea* and belongs to the same branch. The genome assembly and analysis of the cp genome of *E. fortunei* will facilitate the identification, taxonomy, and utilization of *E. fortunei* as well as provide more accurate evidence for the taxonomic identification and localization of Asteraceae plants.

## 1. Introduction

Asteraceae is the second-largest family of the plant kingdom, with a complicated taxonomy and the largest eudicots, consisting of 13 subfamilies, 1689 genera, and 32,913 species [1]. The Asteraceae family is distributed among all continents except Antarctica. *Eupatorium* is a genus of the Asteraceae family, consisting of approximately 36 to 60 flowering plant species [2]. *Eupatorium fortunei* Turcz is a perennial herb that grows on roadsides, thickets, and ravines. The origin of *E. fortunei* is distributed among different provinces of China, including Shandong, Jiangsu, Hubei, Hunan, Yunnan, Sichuan, and a small amount in Japan, Korea, and other countries. It mainly contains coumarin, volatile oil, o-coumaric acid, triterpenoids, and muscadine hydroquinone [3]. The available literature shows that *E. fortunei* has a long history of herbal medicine that exhibits potential antitumor and anti-inflammatory properties, immune regulation of influenza in the body, and the ability to cure heat exhaustion. According to traditional Chinese medicine (TCM), *E. fortunei* has pharmacological effects, such as dampness, strengthening the stomach, resolving dampness, relieving summer heat, and improving spleen functions and appetite [3].

Due to the medicinal importance of *E. fortunei*, it is noteworthy to understand the genetic architecture of its attributes for potential medical use. With the recent development in high-throughput sequencing technology, it has become more interesting to study the cp genomes of both model and nonmodel plants [4]. The chloroplast is a unique organelle of plant cells distinguished by its double-layer membrane and thylakoid structures. It serves as the main site for photosynthesis and efficiently converts light energy to chemical energy through redox reactions. The chloroplast genome ranges from 120 to 180 kb in higher plants [5,6]. Furthermore, it has a lower molecular weight and numerous copies [7]. The circular chloroplast genome has a small single-copy region (SSC), a large single-copy region (LSC), and a pair of inverted regions (IRs) [8,9]. The first chloroplast genome was reported in liverwort [10] followed by tobacco [11].

The rapid development in next-generation sequencing (NGS) technologies, i.e., the Illumina GenomeAnalyzer and Roche/454 GS FLX, has made cp genome sequencing efficient and economical. So far, 4100 plant cp genomes have been published and are available in public repositories, e.g., the NCBI database. In addition, cp genomes contain several functional genes that can be used for species identification and evolutionary studies, which are widely adopted and accepted by researchers [12,13]. Moreover, the cp genome not only determines previously reported phylogeny but also increases the accuracy of phylogenetic trees, thus making the whole sequence of the cp genome a valuable tool for studying molecular phylogeny and ecology [14]. The interspecific relationships within the genus *Eupatorium* and the evolutionary relationships to *E. fortunei* were investigated. Moreover, prior research findings on the Asteraceae family serve as the basis for the present study. To analyze the phylogenetic position and genetic background of *E. fortunei*, we sequenced DNA of the *E. fortunei* species. Furthermore, the complete cp genome sequence of *E. fortunei* was assembled, analyzed, and compared with the cp genomes of Eupatorieae to explore its phylogenetic relationships and provide new insights into the taxonomy and phylogenetic analysis of *Eupatorium*.

## 2. Materials and Methods

### 2.1. Plant Material and DNA Extraction

Fresh leaf material of *E. fortunei* was collected from Yangmingshan National Forest Park, Yongzhou City, Hunan Province, China (26°04′31.8″ N, 111°55′33.8″ E) in May 2021. A total of 6.214 g of fresh plant leaves were taken for the extraction of genomic DNA, and the fresh leaves were immersed in liquid nitrogen and later ground into a fine powder using mortar and pestle. Genomic DNA was extracted using the Plant Genome Rapid Extraction Kit, and then DNA concentration and purity were determined using a NanoDrop 2000 Ultra Micro UV Spectrophotometer [15,16].

### 2.2. DNA Sequencing and Assembly

DNA sequencing was performed at Benagen Technology Services Limited (Wuhan, China). Genomic DNA was detected and collected by 1% agarose gel electrophoresis; the OD_260/280_ value was 1.92, and 1.0 µg of DNA was used for shotgun library construction, and high-throughput sequencing was performed on the Illumina NovaSeq6000sequencing platform. The raw image data files obtained from sequencing were transformed into raw data stored in FASTQ file format (raw reads) by base calling analysis. The raw data were then filtered for low-quality sequences, splice sequences, etc. Quality control of raw sequence reads was carried out using FastQC and Cutadapt using Burrows–Wheeler Aligner’s sequence comparison tool and Samtools toolkit to obtain clean reads [17]. Data were stored in FASTQ format for subsequent analysis and to ensure the reliability of the results.

Presplicing of the genome was performed using the NOVOPlasty V4.2 (https://github.com/ndierckx/novoplasty (accessed on 5 December 2021)) software, and splicing results were analyzed using Blastn and compared on NCBI database to select reference sequences for subsequent genome assembly [18,19]. The collinearity analysis was undertaken on the prespliced results files using the Nucmer command in MUMmer to determine reference sequences’ relative positions and orientations in the genome to continue the construction of the cp genome sequence [19,20]. Moreover, we also verified the correction of the cp genome assembly using the software Getorganelle [21], and the results affirmed whether they joined into a loop to obtain the complete cp genome sequence.

### 2.3. Chloroplast Genome Annotation

Using the chloroplast annotation tool GeSeq (https://chlorobox.mpimp-golm.mpg.de/geseq.html (accessed on 5 December 2021)) [22], the preliminary annotation files of *E. fortunei* cp genome was manually corrected using software Notepad++ and Geneious 8.0.4 [23] for each gene where necessary. The annotation files of cp genome sequence were uploaded to OrganellarGenomeDRAW (OGDRAW, http://ogdraw.mpimp-golm.mpg.de (accessed on 5 December 2021)) with default setting to determine the order of gene alignment and the position of the IRs with the LSC region and the SSC region [24], and finally to generate the physical mapping of the cyclic cp genome of *E. fortunei*. The cp genome sequence and annotation files of *E. fortunei* were submitted to the NCBI database (https://www.ncbi.nlm.nih.gov/ (accessed on 5 December 2021)) to obtain the genome accession number.

### 2.4. SSRs, Long Repeat Sequence, and Codon Usage Analysis

The microsatellite identification tool MISA (https://webblast.ipk-gatersleben.de/misa/ (accessed on 1 January 2022)) was used to identify and localize potential single sequence repeat (SSR) sites in the complete cp genome sequence [25]. Parameters setting: the minimum repeat numbers were set as 10 units for mononucleotides, 6 units for dinucleotides, and five for tri-, tetra-, penta-, and hexanucleotides whereas the interruptions (max_difference_for_2_SSRs) were set as 100 bp. The REPuter (https://bibiserv.cebitec.uni-bielefeld.de/reputer?id=reputer_manual_manual (accessed on 1 January 2022)) was used for the identification of palindromic, forward, reverse, and complement repeats present in the cp genomes, in which the Hamming distance was set as 3, and the minimum repeat size was 30 bp [26]. Relative synonymous codon usage (RSCU) of protein-coding genes was assessed using the CodonW software (University of Texas, Houston, TX, USA) to analyze the codon usage preference.

### 2.5. Genome Comparison and Structural Analysis

We used IRScope software (https://irScope.shinyapps.io/Irapp/ (accessed on 3 January 2022)) [27] to generate a comparison diagram of the inverted repeat region (IR) boundary to quantify the gene and neighboring gene characteristics at each border region (LSC-IRa, IRa-SSC, SSC-IRb, IRb-LSC).

The cp genome sequences were aligned and visualized using the online software mVISTA (https://genome.lbl.gov/vista/mvista/submit.shtml (accessed on 3 January 2022)). Then, Shuffle-LAGAN global alignment mode was selected, and other parameters were set at default values to find gene rearrangements and inversions. Nucleotide divergence of Eupatorieae species was calculated based on nucleotide diversity value by employing DnaSP v6 software [28].

### 2.6. Phylogenetic Analysis

To analyze the phylogenetic relationship of *Eupatorium* species within Eupatorieae, multiple sequence alignments were generated using the whole cp genome sequences of 20 Eupatorieae species, and the cp genome sequences of 19 species were downloaded from the NCBI database. In addition, we selected two other species, *Campanula takesimana* and *Platycodon grandiflorus* from Campanulaceae as outgroups, and the detailed information is listed in Table S1. Multiple sequence alignment of nucleotide sequences was executed using MAFFT (v7.481) [29], and the maximum likelihood (ML) method based on single-copy genes (CDS) in MEGA 7.0.14 and RAxML v 8.2.12 [30] was conducted for phylogenetic analysis with default parameter settings and 1000 bootstrap repeats [31]. The ML tree was constructed using RAxML (v.8.2.12) and applied with 1000 bootstrap repeats at each branch node.

## 3. Results

### 3.1. Genome Sequencing and Assembly

The *E. fortunei* cp genome coverage was 100% using the high-throughput sequencing platform Illumina NovaSeq6000. The raw short sequence data file (sequenced reads) of the double-ended Illumina reads obtained from sequencing were approximately 9.4 GB, which contained information on the bases of the sequences (reads) and their corresponding sequencing quality information. The total number of sequences was 26,889,364, and the average length was 150 bp. Statistics of the sequencing data showed that the number of bases was 4,033,404,600 bp. Zero sequences were flagged as poor quality, and the base error rate was 2.67%, the GC content was 36.76%, and the percentage of bases with Phred values greater than 20 and 30 was 97.4% and 92.55% of the overall bases. To ensure the quality of information analysis, the reads with connectors and low quality were removed. After data filtering, the clean data volume was 320 Mb reads file, which was utilized for subsequent information analysis. After preliminary splicing, three valid contigs were obtained: contig 01 with 133,092 bp, contig 02 with 19,561 bp, and contig 03 with 19,309 bp. The complete cp genome of *E. fortunei* was obtained after genome fragment splicing and gap filling.

### 3.2. Basic Structural Properties of the Chloroplast Genome

**Figure 1:**
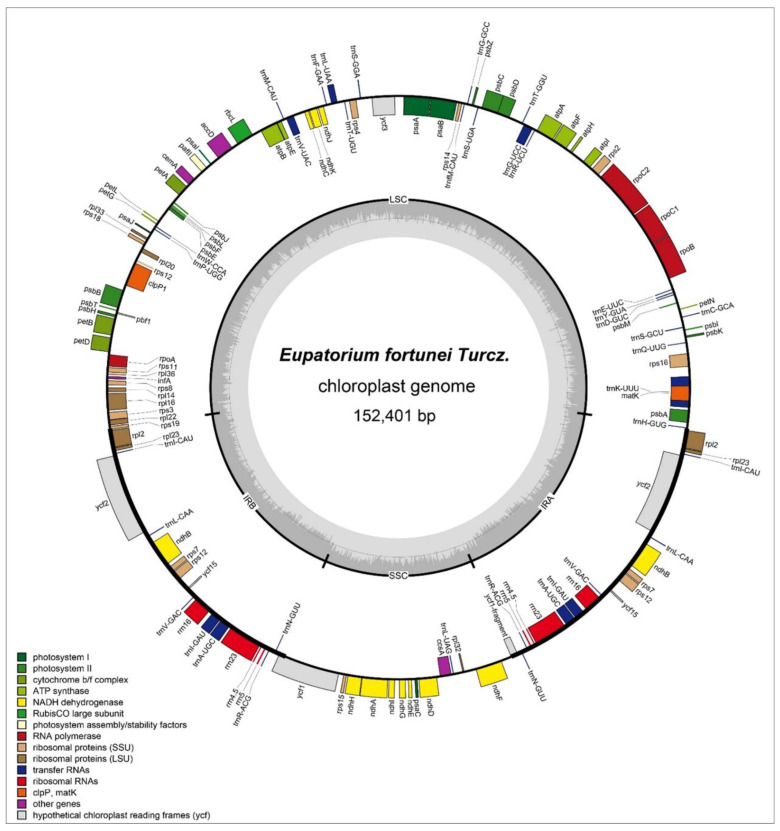
The chloroplast genome of *E. fortunei.* Genes located outside the outer rim are transcribed in a counterclockwise direction whereas genes inside the outer rim are transcribed in a clockwise direction. The colored bars indicate the known different functional groups. The dashed gray area in the inner circle shows the percentage GC contents of the corresponding genes. LSC, SSC, and IR denote large single copy, small single copy, and inverted repeat, respectively.

The cp genomes exhibited a typical circular quadripartite structure. The total length of the three species of section Eupatorieae cp genomes ranged in size from 150,698 bp (*Ageratina adenophora*) to 152,401 bp (*E. fortunei*) (Figure 1, Table 1). The difference in cp genome length between the largest and smallest genomes was 1703 bp. In terms of length, the cp genome of *E. fortunei* was 1.16 kb longer than *Stevia* sp. *Oliveira* 769 whereas it was 0.6 kb, 0.4 kb, and 0.88 kb longer than *Mikania burchellii*, *Mikania sylvatica*, and *Litothamnus nitidus*, respectively, however, only 32 bp longer than *Ageratina fastigiata*.

The cp genomes contained four characteristic regions in all three Eupatorieae species. The length of the LSC region ranged from 83,032–84,829 bp, and the SSC region ranged between 18,309–34,967 bp; in all cp genomes selected, these two regions were separated by two IR regions (30,902–50,060 bp). Furthermore, the coding region ranged from 73,554 bp to 78,621 bp, and the noncoding region sequence lengths ranged from 73,748 bp to 77,144 bp. The overall GC content was observed between 37.46–37.59%. In addition, the GC content was unevenly distributed among the regions of the cp genome, with high GC content in the IR region, accounting for 38.48–43.06%, and a relatively low GC content in the LSC and SSC regions of 35.59–35.74% and 31.15–40.73%, respectively (Table 1).

The three cp genomes consisted of 133–136 genes, including 87–86 protein-coding genes (PCGs), eight rRNA-coding genes and 37 tRNA-coding genes, and 1–5 pseudogenes (Table 2). One pseudogene (*ycf1*) was found in *E. fortunei*. In Magnoliophyta, the structure and sequence composition of the cp genomes were highly conserved. The gene composition of *E. fortunei* was the same as that of most Magnoliophyta plant cp genomes. No major gene gain or loss was found, and it had the typical structure of Magnoliophyta plant cp genomes [32].

In the *E. fortunei* plant, the cp genomes sequenced were all encoded of 115 single-copy and 18 duplicated genes, which is similar to that of *A. fastigiata*. Duplicated genes, including seven PCGs (*rpl23*, *rpl2*, *rps7*, *rps12*, *ndhB*, *ycf2*, *ycf15*), seven tRNA genes (*trnA-UGC*, *trnI-CAU*, *trnL-CAA*, *trnI-GAU*, *trnN-GUU*, *trnR-ACG*, and *trnV-GAC*) genes, and four rRNA genes, were also present in the IR region in two copies (Table 1). Based on function, the cp genes were classified into three categories: category one includes 74 genes related to transcription and translation; category two contains 45 genes related to photosynthesis, and category three includes 14 genes associated with the biosynthesis of substances, such as amino acids and fatty acids and some genes with unknown function (Table 2).

### 3.3. SSRs Analysis 

Simple sequence repeats (SSRs) are efficient molecular markers with the advantages of abundance, high reproducibility, codominant inheritance, uniparental inheritance, and relative conservation, making them the best fit for species identification and evaluation of genetic variation at both the population and individual levels [33].

**Figure 2:**
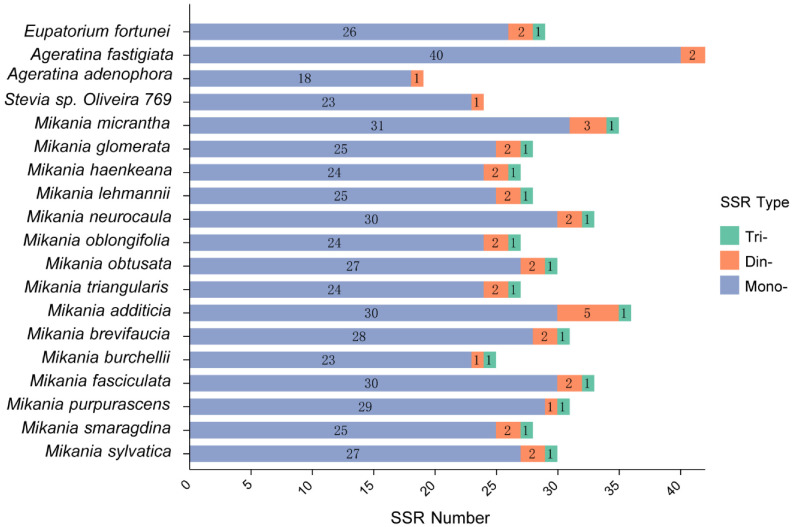
Analysis of simple sequence repeat (SSR) in the chloroplast genomes of 20 Eupatorieae species. The *x*-axis represents the numbers of SSR in each chloroplast genome. The *y*-axis represents 20 Eupatorieae species. Mono-, mono-nucleotide repeat; Di-, dinucleotide repeat; Tri-, trinucleotide repeat; Tetra-, tetranucleotide repeat.

**Figure 3:**
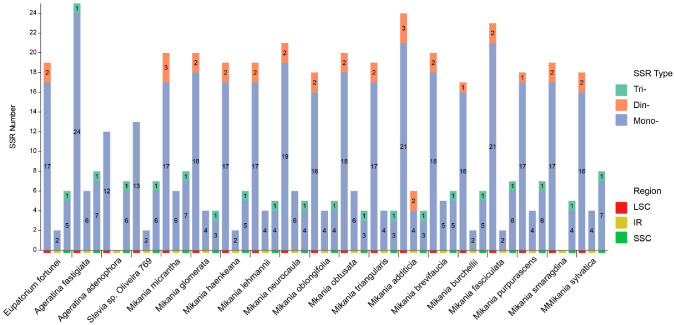
Distribution of SSRs in various regions of 19 Eupatorieae species. The *x*-axis represents the 20 Eupatorieae species and the position of each SSR in the chloroplast genome. The *y*-axis represents the number of SSRs in each chloroplast genome. Mono-, mononucleotide repeat; Di-, dinucleotide repeat; Tri-, trinucleotide repeat; Tetra-, tetranucleotide repeat.

To explore the distribution and differences of SSRs among 20 Eupatorieae species, we detected SSRs in the cp genomes of these species using MISA. This study analyzed the number, type, and spatial distribution of SSRs in 20 Eupatorieae cp genomes (Figure 2). The total SSR loci in cp genomes ranged from 19 in *A. adenophora* to 42 in *A. fastigiata*. SSRs were disproportionately spaced for the cp genomes; the SSRs frequency of the LSC region was significantly higher than that of the IRs and SSC regions (Figure 3). Most of the SSRs were distributed in LSC (66.79%) followed by SSC (20.55%), and IRs accounted for 12.67%. Since the *P. clematidea* tetrameric structures of IRb and IRa are not fully complementary in the reverse direction, the species were not identified in the following analysis involving tetrameric structures. Most of the SSR sequences in the cp genome of 20 Eupatorieae plants were composed of mononucleotide and dinucleotide repeated units. The number of mononucleotide repeats ranged from 18 in *A. adenophora* to 40 in *A. fastigiata*. These were followed by dinucleotides (1–5) and trinucleotides (1–2). The different types of SSRs can be seen with their unique features and can be used as cp markers.

In the *E. fortunei* species, 13 SSRs were distributed in the intergenic spacer (IGS), accounting for 44.8% of the total. A total of 10 SSRs appeared in the coding regions of *trnK-UUU*, *rpoB*, *rpoC1*, *trnT-GGU*, *rpoA*, and *ycf1*, with five SSRs in the coding region of the *ycf1* gene and another six SSRs located in the intron regions of *rps16*, *rpoC1*, *rps12* and *clpP1* (Table 3).

### 3.4. Long Repeat Analysis

**Figure 4:**
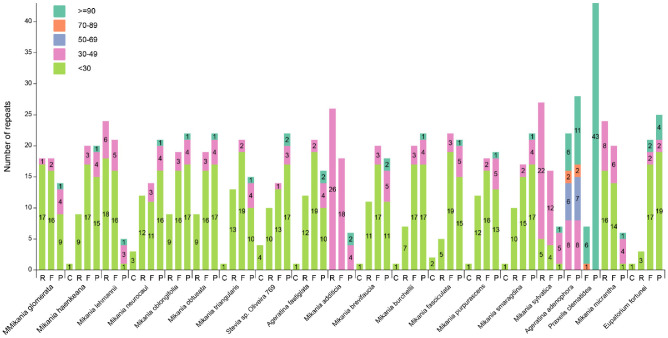
Analysis of long repeats in 20 different cp genomes. The *x*-axis represents the long repeat types, and the *y*-axis represents the numbers of corresponding long repeat types. F—forward repeat; R—reverse repeat; C—complement repeat; P—palindromic repeat. Different colors indicate different sizes of long repeat sequences.

A repeat sequence can be divided into five types according to length (<30, 30–49, 50–69, 70–89, ≥90 bp), and there are four types according to the type of repetition: forward (F), reverse (R), complement (C), and palindromic (P). In this study, we analyzed the long repeat sequence of the complete cp genomes of 20 species and found that each species had 50 long repeats, respectively (Figure 4). Among them, *P. clematidea* and *A. adenophora* had the minimum number of repetition types, the former including seven forward repeats, and 43 palindromic repeats and the latter including 22 forward and 28 palindromic repeats. *P. clematidea* contains the most palindromic repeats and the fewest forward repeats. In addition, *E. fortunei* has one complement repeat, 21 forward, 25 palindromic, and three reverse repeats.

Of the 20 species, *Stevia* sp. *Oliveira* 769 contained the highest number of repeat sequences of ≤30 bp in length. All of the species contained ≥90 bp repeat sequences; two species, *A. adenophora* (4) and *P. clematidea* (1), contained repeat sequences of 70–89 bp, and one species, *A. adenophora* (13), contained repeat sequences of 50–69 bp. All 20 species showed the maximum number of repeats ≤ 30 bp and 30–49 bp in length while one species did not contain 30–49 bp and ≤30 bp repeat sequences. Among them, ≥90 bp long repeat sequences in *P. clematidea* (49) and *A. adenophora* (17) were the most abundant. In addition, other species contained only one or two repeat sequences with a length of ≥90 bp.

### 3.5. Analysis of Codon Preference

**Figure 5:**
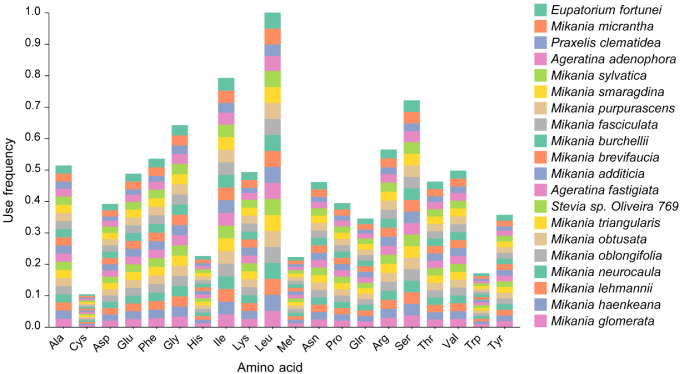
Frequency of amino acids encoded in the coding region by codons of chloroplast genes from 20 Eupatorieae species. Frequency: the relative frequency of codon (amino acid) used in different species. That is, the frequency of other codons relative to Leu using Leucine (Leu) as a benchmark.

Codon preference is the uneven utilization of synonymous codons encoding the same amino acid in an organism [32]. Developed during the long-term evolution of organisms, codon preference has a complex set of synthesis mechanisms [34]. The following Figure 5 shows the codon preference analysis of the genes present in the 20 Eupatorieae species.

In the amino acid encoded by the codon, the codon for Leucine (Leu) was used most frequently. Among the 20 species, the codon for Leu was encoded 2608 times in *Mikania sylvatica* and 2606 times in *Mikania brevifaucia*; the least frequently coded amino acid was cysteine (Cys) with a frequency of 205 for *P. clematidea*, 206 for *A. denophora* (260 times), and 265 for *Mikania micrantha.* Of the 20 amino acids encoded, Leu (5.10–3.71%) was the most frequent amino acid followed by Isoleucine (Ile) with 3.09–4.07% and Serine (Ser) with 2.51–3.68% while Cys (0.40–0.53%) was the least.

### 3.6. Expansion and Contraction of Border Regions

**Figure 6:**
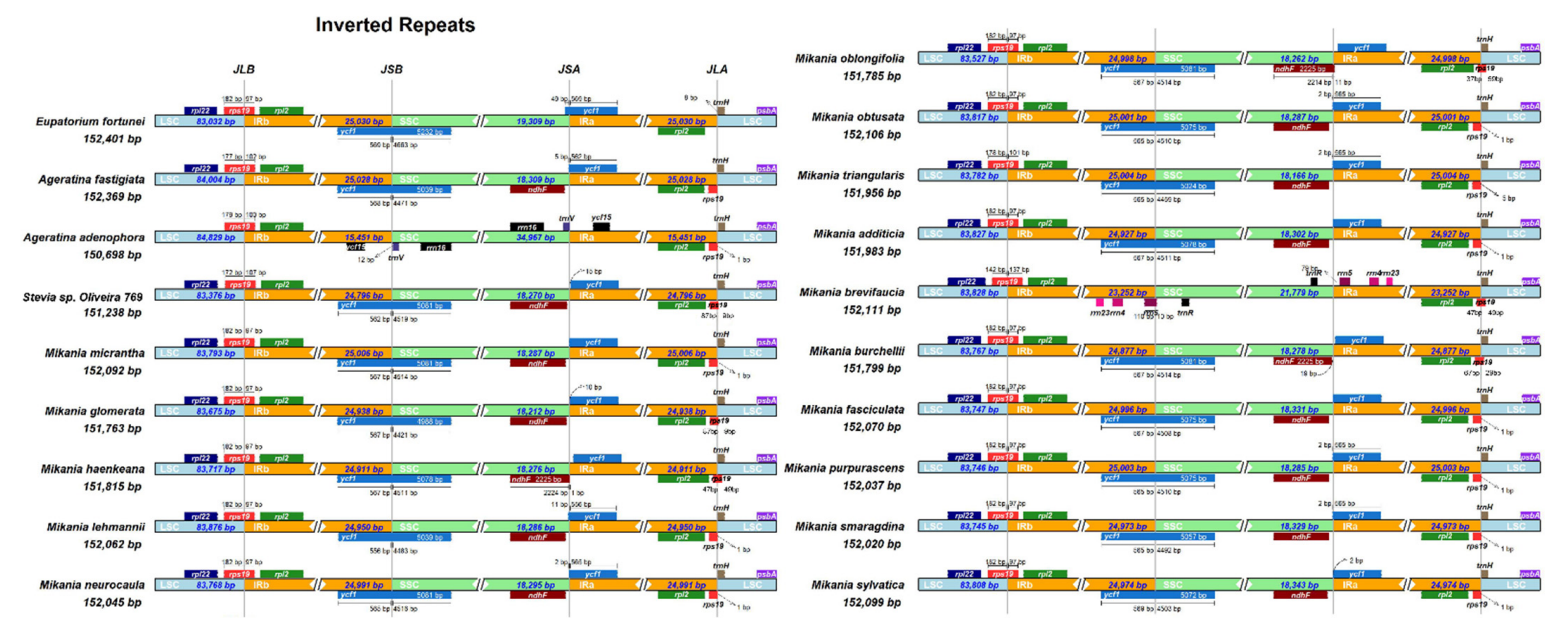
Comparison of LLC, IR, and SSC border regions among 19 Eupatorieae chloroplast genomes. (The light blue, yellow, and grass green blocks represent the LSC, IR, and SSC regions, respectively. JSA: junction of the SSC and the IRA; JLB: junction of the LSC and the IRB; JSB: junction of the SSC and the IRB. Boxes above or below the main line indicate genes adjacent to borders.)

The expansion and contraction of IRs may cause alterations in the structural variations of cp genomes, an important factor for variation in an cp genome, whereas the length of its specific position and interval is an important evolutionary trait between species [32]. Therefore, comparing the borders and boundary genes of Eupatorieae species to those of *E. fortunei*, the expansion and contraction diversity of the connected regions have been analyzed (Figure 6). The LSC-IRb, SSC-IRb, SSC-IRa, and LSC-IRa boundaries in the 19 cp genomes were located in *rps19*, *ycf1*, *ndhF,* and *ycf1*, *rpl2*, and *trnH*, contained six genes, respectively. The cp genomes of Eupatorieae species were relatively conserved in terms of gene arrangement, structure, and number of genes.

The LSC-IRb cp genome boundaries were mostly similar. The *rps19* gene crossed the LSC/IRb border with the larger part located in the LSC region, which showed various degrees of contraction and expansion at the LSC/IRb boundary. In addition, the *rps19* gene in the cp genomes of most species exhibited 89 bp protrusion in the IRb region. In addition, a IRb-SSC junction was located in the *ycf1* region in the cp genomes of all 19 species and extended a different length into the SSC region. In contrast, *ycf1* was mainly located in the SSC region, ranging from 4988 bp to 5232 bp in *Mikania glomerata* and *E. fortunei*, respectively, while *Mikania brevifaucia* did not contain the *ycf1* gene. As the *ycf1* gene straddled the SSC/IRa boundary, a pseudogene was generated in the IRb region.

Further analysis revealed that the *E. fortunei* LSC region was smaller among the 19 Eupatorieae chloroplasts. In contrast, the IR region was larger, indicating that the *E. fortunei* IR region expanded and altered the sequence length of its entire genome. The 19 species showed that the variations in the IR/SC boundary region in the cp genomes were responsible for the differences in the lengths of the four regions and whole-genome sequences.

### 3.7. Sequence Diversity Analysis of Chloroplast Genomes

**Figure 7:**
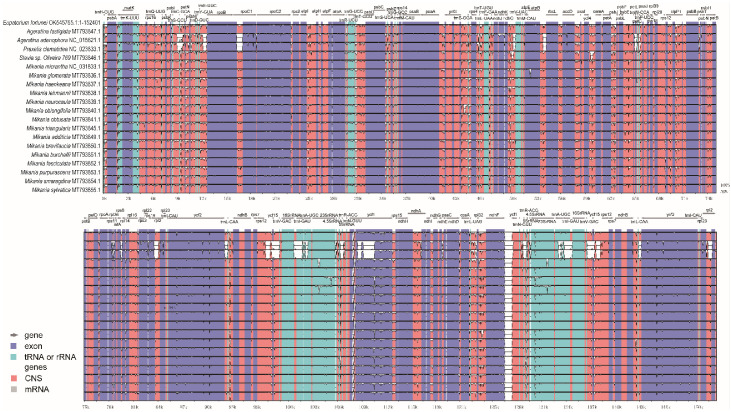
Percent identity plot for comparison of 19 Eupatorieae cp genomes using mVISTA. The *x*-axis represents the base sequence, and on the *y*-axis, percentage of sequence identity was shown between 50% and 100%. In contrast, coding exons and UTRs are marked with different colored rectangles. Conserved regions (defined below) are highlighted under the curves, red indicates conserved noncoding regions, blue indicates conserved exons, conserved UTRs are turquoise, and the direction of gene transcription is indicated by gray arrows. The pink bars represent noncoding sequences (CNS) while the purple bars represent exons.

The sequence similarity of 20 Eupatorieae chloroplasts was analyzed via the mVISTA whole gene sequence alignment tool, which revealed high sequence similarity between cp genomes, indicating that genome structure was relatively conserved at the gene sequence level (Figure 7). Notably, the IR regions exhibited less divergence than the SSC and LSC regions. In particular, PCGs had a high similarity of more than 95%. The gene spacer region of chloroplasts has applications for species phylogeny, molecular identification, and molecular barcoding. Although the 20 cp genomes showed similar patterns, several significant nucleotide variations were detected in the coding regions. For instance, the coding regions such as *rpoB*, *rpoC2*, *atpI*, *psaB*, *accD*, *rps3*, *ycf2*, *ycf1*, and *ndhF* showed a high level of nucleotide variation. Among these coding regions, *ndhD-ccsA*, *psbI-trnS*, *trnH-psbA*, *ndhF-ycf1*, and *ndhI-ndhG* were significantly different with a sequence similarity lower than 85%. The results revealed data useful for identifying candidate sequence loci of the new Eupatorieae plants for phylogenetic studies.

### 3.8. Nucleotide Polymorphism Analysis

**Figure 8:**
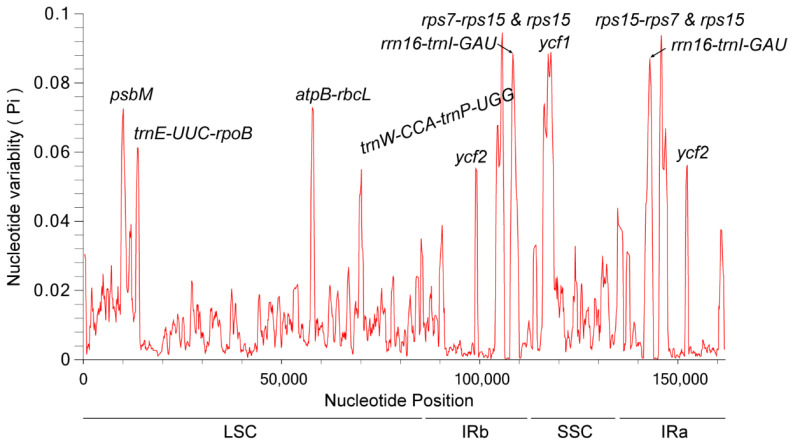
Sliding window analysis for the nucleotide diversity (π) of the whole chloroplast genomes for Eupatorieae species. *X*-axis: position of the midpoint of a window, *Y*-axis: nucleotide diversity (pi) of each window.

The recognition of highly variable sites in the whole cp genomes can be used as molecular markers for species identification and phylogenetic studies [35]. This study screened the highly variable sites of 20 Eupatorieae species by sliding window analysis, and 11 high variable regions of 20 cp gnomes were found (Figure 8). The nucleotide diversity values (Pi) ranged from 0 to 0.09451 in the 20 Eupatorieae species. The *rps7-rps15* and *rps15* regions had the highest nucleotide diversity (0.09451) followed by *ycf1* (0.08885) and *rrn16-trnl-GAU* (0.08847).

The highly variable sites were distributed in the LSC region (*psbM*, *trnE-UUC-rpoB*, *atpB-rbcL*, *trnW-CCA-trnP-UGG*), IRb region (*ycf2*, *rps7-rps15 & rps15*, *rrn16-trnl-GAU*), SSC region (*ycf1*), and IRa region (*rps15-rps7* & *rps15*, *rrn16-trnl-GAU*, *ycf2*). Their nucleotide variability (π > 0.06) was significantly higher than that of other regions. The LSC region had the most variation sites followed by the IRb and IRa regions. In contrast, the SSC region had relatively few variation sites. The distribution of pi values was significantly lower than in LSC and IRs regions; it also revealed that in the cp genomes, the SSC region variation was conservative compared to LSC and IRs regions. This indicates that the degree of nucleotide variability varies among the cp genomes, and the LSC region is a high-frequency region for variation. Analysis of the variant sites in all 20 Eupatorieae species may help in the screening of more suitable molecular identification markers for Eupatorieae species.

**Figure 9:**
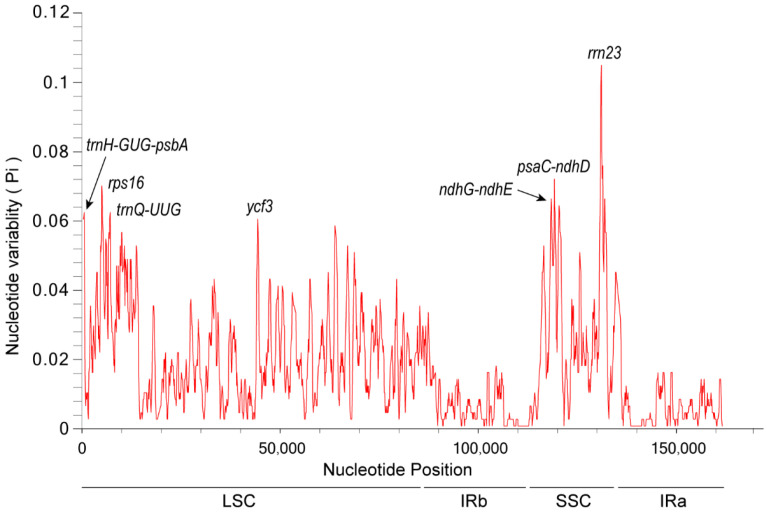
Sliding window analysis of nucleotide diversity: position of the midpoint of a window, *Y*-axis: nucleotide diversity (pi) of each window.

In addition, the nucleotide polymorphisms of *E. fortunei* and the closely related species *P. clematidea* were analyzed (Figure 9). The results showed that their pi values ranged from 0 to 0.10800, and a total of seven highly variable sites were found (*trnH-GUG-psbA*, *rps16*, *trnQ-UUG*, *ycf3*, *ndhG-ndhE*, *psaC-ndhD*, *rrn23*), all with pi values greater than 0.06. The LSC and SSC regions had high variability, with the highest nucleotide diversity in *rrn23* (0.10800) followed by *psaC-ndhD* (0.07400). 

### 3.9. Phylogenetic Analysis

**Figure 10:**
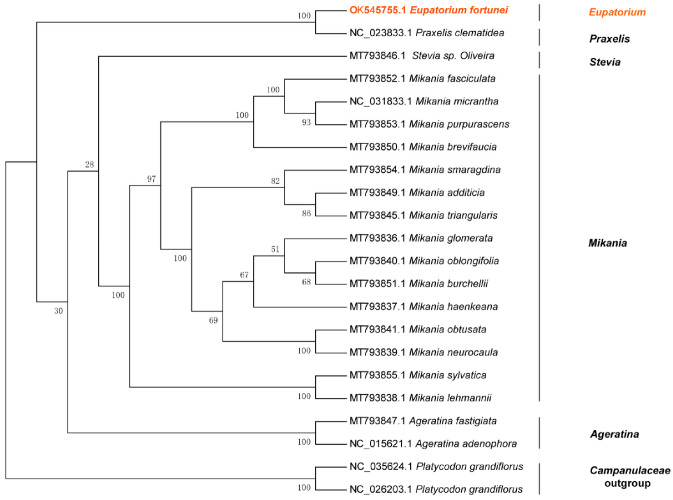
Phylogenetic relationship of 22 species inferred from maximum likelihood (ML). ML tree constructed based on 19 protein-coding genes (CDS) and the species *E. fortunei* is marked in red.

**Figure 11:**
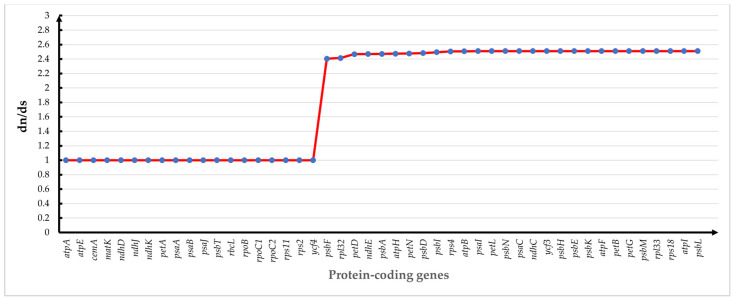
Selective pressure analysis of 51 common protein-coding genes in 22 species cp genomes.

The maximum likelihood (ML) was used to construct phylogenetic trees based on one data set (51 single-copy genes) from 22 species to assess the phylogenetic relationship and the loci position of *E. fortunei* (Figure 10). We performed ka/ks analysis on 51 coding genes shared by 22 species, and the ratio of synonymous substitution rate (dS) to nonsynonymous substitution rate (dN) (ω = dN/dS) was calculated using the CODEML module in PAML for 51 protein-coding genes shared by 22 species. In ka/ks analysis, if ω > 1, it is positive selection (positive selection); if ω = 1, it is neutral selection (neutral selection); if ω < 1, it is purifying selection (purifying selection). The results are shown in Figure 11. 19 genes with ω value of 1 were screened, and the CDS sequences of these genes were used to construct the evolutionary tree. The ML tree topology-constructed bootstrap support values were detected on most nodes, and two outgroup species (*P. grandiflorus* and *C. takesimana*) were independent. In addition, 15 *Mikania* species were clustered into a single large clade with bootstrap support values of 100%. The phylogenetic tree was consistent with the traditional morphology-based taxonomy of Asteraceae species. *E. fortunei* and *P. clematidea* were clustered together and constructed a well-supported monophyletic evolutionary branch. *Stevia* sp. *Oliveira* 769 and *E. fortunei* exhibited a sister relationship, indicating the close relationship between the three Eupatorieae species. In comparison, *E. fortunei* is more closely related to *P. clematidea*, a member of the same branch, with a bootstrap value of 100%. As a result, it can be deduced that they are the most closely related species. Our phylogenetic analysis clarified the position of *E. fortunei* within Eupatorieae, and it is worth mentioning that this is the first time to clarify the position of *Eupatorium* in Asteraceae.

## 4. Discussion

Asteraceae plants are found around the world and less dispersed in the tropics. In the second expression of diversity variation, studies of Asteraceae plants revealed different alterations in inflorescence morphology and chromosome number [36]. From a functional point of view, Asteraceae plants include important economic food crops [1], herbs [37], ornamental flowering plants, and some invasive species that can have a huge impact on the ecological environment, such as *P. clematidea*, *A. adenophora*, and *Pityosis* [2,38,39]. Asteraceae plants are diverse in species, similar in phenotype, relatively late in origin, in a strong differentiation stage, and exhibit many intermediate links in evolution. The division of their family level and systematic research have posed significant challenges, and the systematic relationship within their family has always been a contentious issue in botany research.

The highly conserved nature and low evolutionary rate of the cp genome make it a hotspot for phylogenetic studies among different species [32], thus making the whole sequence of the cp genome a valuable tool for studying molecular phylogeny and ecology [14]. From the cp genome, the genetic relationships and evolutionary characteristics of medicinal plants were investigated with greater precision. In the present study, we sequenced the whole cp genome of *E. fortunei* using NGS technology and obtained the complete cp genome sequence of *E. fortunei* after splicing, assembly, and gap filling. Worth noting, it is the first study that reported a complete cp genome of *Eupatorium.* In comparison to other Eupatorieae species of the NCBI genomic database, we found that Eupatorieae species have similar cp genome structure, genomic base GC content, and gene composition. Reflecting the stable cp genome structure and low overall evolutionary rate of Eupatorieae, it has typical Magnoliophyta plant characteristics. [34]. The *E. fortunei* cp genome was determined to be 152,401 bp in length, similar to the other reported Eupatorieae plants cp genomes; the *E. catarium* cp genome is 151,410 bp in length [2]. The *A. adenophora* cp genome length is 150,698 bp [38]. However, there were differences in genome size, indicating genetic differences. The cp genome of *E. fortunei* has a double-stranded cyclic tetrad structure, and the cp genome structure and gene types were highly conserved, which shows that substantial conservation in genomic structure and gene capacity when compared to closely related species. The boundaries of SSC/IR and LSC/IR were compared and found to be different in the cp genomes among Eupatorieae species. Overall, the selected species were more conserved in the LSC/IRb, IRa/LSC, IRb/SSC border regions. The IRb/IRa border regions showed variations that may be due to the contraction and expansion of the outer regions [33]. It was also observed that both IRb/SSC and IRa/LSC regions were the main cause of sequence length differences in the cp genomes [40], and this phenomenon was also found in most of the Magnoliophyta cp genomes [41].

The *E. fortunei* cp genome has a codon preference for A and T, especially at the second and third positions of the codon. Microsatellites can be divided into mono-, di-, tri-, tetra-, penta-, and hexanucleotide repeats. *Eupatorium* and *Mikania* show highly uniform characteristics, respectively, while *Ageratina* differs from them. In the proportion of A/T motif and mononucleotide repeats, *E. fortunei* is closer to the characteristics of Mikania glomerata. In the SSR multidirectional comparison, *E. fortunei* and *A. fastigiata* have notable differences. The locations of SSRs have functional roles in the genomes, including gene regulation and evolution. In the *E. fortunei* cp genome, SSRs are mainly found in mononucleotide repeats located at the LSC region. Identifying SSRs will help promote population genetics studies in measuring population genetic diversity, gene origins, and intraspecific and interspecific variation [42,43]. Whole sequence alignment of the cp sequences of Eupatorieae plants using mVISTA software indicates that coding regions were more conserved than noncoding regions.

Due to the overall conserved nature of the cp genomes, it is common to screen for highly variable regions in the chloroplast, such as *matK*, *trnH-psbA*, and *rbcL*. Using cp genome-wide information, high variability regions in the cp genome can be screened comprehensively and accurately. In a comparative study of 20 species of Eupatorieae, nucleotide polymorphism of sequences performed by sliding window analysis revealed the commonly used cp molecular barcode regions. Meanwhile, the *PsbM*, *rps15-rps7* and *rps15*, *rrn16-trnl-GAU*, and *ycf1* regions showed significantly higher nucleotide variability than other regions. Therefore, the phylogeny and pedigree geography of *Eupatorium* plants in these regions could be prioritized in the selection of cp molecular markers in future research. Based on the correlation of all cp genomes, the taxonomic position and evolutionary relationship of *E. fortunei* were analyzed by comparing multiple Eupatorieae plants. In the current study, the results indicated that *E. fortunei* was most closely related to *P. clematidea* followed by *Stevia* sp and *A. fastigiata*, respectively. However, the long repeat fragment types differed significantly between the *E. fortunei* and *P. clematidea*. Comparative analysis of genomic level was performed in conjunction with cp genomes of 20 species of Eupatorieae, providing genetic resources for the development of *E. fortunei* chloroplast-based molecular markers, which may be useful for the phylogeny of Eupatorieae and even Asteraceae. In the near future, large-scale sequencing of organelle genomes will curb the current limitations of using short sequences for phylogenetic analysis and combined with the genetic information in the nuclear genome, will better address the phylogenetic study of *Eupatorium*.

## 5. Conclusions

In this study, the architecture of the *E. fortunei* cp genome, including its basic features, repeat sequences, SSRs, codon preferences, and phylogenetic relationships, were described comprehensively. Then, the cp genome of *E. fortunei* was compared with 19 other Eupatorieae species. The cp genome of *E. fortunei* had a typical quadripartite structure and 133 functional genes were annotated, including 87 protein-coding genes, 37 tRNA genes, eight rRNA genes, and one pseudogene. The genome size and genomic contents are similar to other Eupatorieae species. In total, 11 highly variable regions were chosen as potential molecular markers for the Eupatorieae species, which could be employed in population genetic studies. These findings enrich our knowledge of cp genomics and the genetic diversity of *E. fortunei* and lay a strong foundation for further studies on molecular marker development, phylogenetic analysis, population studies, and cp genome engineering.

## Figures and Tables

**Table 1:** The basic characteristics of the chloroplast genomes of three Eupatorieae species.

Species Names	*E. fortunei* OK545755.1	*A. fastigiata* MT793847.1	*A. adenophora* NC_015621.1
Total length (bp)	152,401	152,369	150,698
LSC length (bp)	83,032	84,004	84,829
SSC length (bp)	19,309	18,309	34,967
IR length (bp)	50,060	50,056	30,902
Coding length (bp)	78,396	78,621	73,554
Noncoding length (bp)	74,005	73,748	77,144
Total number of genes	133	134	136
Protein-coding genes (duplicated)	87 (7)	87 (7)	86 (7)
tRNA genes (duplicated)	37 (7)	37 (7)	37 (10)
rRNA genes (duplicated)	8 (4)	8 (4)	8 (4)
Pseudo genes	1	2	5
GC content of genome	37.5884	37.5018	37.4617
GC content of LSC	35.7103	35.5852	35.7424
GC content of SSC	31.4775	31.1540	40.7298
GC content of IR	43.0604	43.0398	38.4830

Note: The number of multicopy genes is in brackets.

**Table 2:** List of genes in the chloroplast genome of *E. fortunei*.

Function	Group of Genes	Name of Genes	Total Number
Self-replication	Large subunit of ribosome	*rpl20,rpl22,rpl32,rpl23(X2),rpl14,rpl33,rpl16,rpl36,rpl2(X2)*	11
	Small subunit of ribosome	*rps11,rps14,rps15,rps16,rps2,rps3,rps18,rps19,rps4,rps7(X2),rps8,rps12(X2)*	14
	DNA-dependent RNA polymerase	*rpoA,rpoB,rpoC1,rpoC2*	4
	rRNA gene	*rrn5(X2),rrn4.5(X2),rrn16(X2),rrn23(X2),*	8
	tRNA gene	*trnR-UCU,trnE-UUC,trnI-CAU(X2),trnS-GGA,trnT-GGU,trnR-ACG(X2),trnV-GAC(X2),trnL-UAA,trnG-GCC,trnD-GUC,trnL-CAA(X2),trnP-UGG,trnM-CAU,trnY-GUA,trnS-GCU,trnW-CCA,trnF-GAA,trnT-UGU,trnS-UGA,trnV-UAC,trnG-UCC,trnL-UAG,trnI-GAU(X2),trnH-GUG,trnN-GUU(X2),trnA-UGC(X2),trnfM-CAU,trnQ-UUG,trnK-UUU,trnC-GCA*	37
Gene for photosynthesis	Subunits of photosystem I	*psaA,psaB,psaC,psaI,psaJ*	5
	Subunits of photosystem II	*psbL,psbZ,psbM,psbA,psbB,psbC,psbD,psbE,psbF,psbT,psbH,psbI,psbJ,psbK,psbN*	15
	Subunits of NADH-dehydrogenase	*ndhG,ndhH,ndhI,ndhJ,ndhK,ndhA,ndhB(X2),ndhC,ndhD,ndhE,ndhF*	12
	Subunits of cytochrome b/f complex	*petL,petA,petN,petB,petD,petG*	6
	Subunit for ATP synthase	*atpI,atpA,atpB,atpE,atpF,atpH*	6
	Large subunit of rubisco	*rbcL*	1
Other genes	Translational initiation factor	*infA*	1
	Maturase	*matK*	1
	Protease	*clpP1*	1
	Envelope membrane protein	*cemA*	1
	Subunit of Acetyl-carboxylase	*accD*	1
	C-type cytochrome synthesis gene	*ccsA*	1
Unknown function	Open reading frames (ORF,ycf)	*ycf1,ycf2(X2),ycf3,ycf4,ycf15(X2),ycf1-fragment*	8

**Table 3:** Distribution of SSRs within *E. fortunei* chloroplast genome.

Number	SSR Type	Size	Start	End	Position
1	(T)10	10	2299	2308	*trnK-UUU*
2	(C)12	12	5418	5429	*rps16* intron
3	(T)10	10	9543	9552	IGS
4	(A)10	10	13,302	13,311	*rpoB*
5	(T)12	12	16,571	16,582	*rpoC1* intron
6	(A)10	10	18,356	18,365	*rpoC1*
7	(T)13	13	24,915	24,927	IGS
8	(TA)6	12	26,593	26,604	IGS
9	(T)14	14	27,969	27,982	IGS
10	(T)10	10	30,842	30,851	*trnT-GGU*
11	(AT)7	14	43,871	43,884	IGS
12	(T)14	14	46,306	46,319	IGS
13	(T)12	12	53,844	53,855	IGS
14	(T)11	11	58,539	58,549	IGS
15	(T)10	10	69,445	69,454	*rps12* intron
16	(T)10	10	69,481	69,490	*clpP1* intron
17	(T)10	10	70,461	70,470	*clpP1* intron
18	(T)10	10	70,625	70,634	*clpP1* intron
19	(T)10	10	77,060	77,069	*rpoA*
20	(T)10	10	79,989	79,998	IGS
21	(A)11	11	106,342	106,352	IGS
22	(GAA)5	15	108,410	108,424	*ycf1*
23	(T)12	12	108,521	108,532	*ycf1*
24	(T)10	10	108,649	108,658	*ycf1*
25	(A)11	11	109,134	109,144	*ycf1*
26	(A)10	10	110,236	110,245	*ycf1*
27	(A)13	13	120,967	120,979	IGS
28	(T)13	13	122,925	122,937	IGS
29	(T)11	11	129,082	129,092	IGS

## Data Availability

The data that support the findings of this study are openly available in GenBank of NCBI at https://www.ncbi.nlm.nih.gov/nuccore/OK545755 (accessed on 20 December 2021). The accession number is OK545755.

## Supplementary Materials

The following are available online at https://www.mdpi.com/article/10.3390/genes14010064/s1, Table S1. Chloroplast genome information table for 22 species in the article.
