# Supplementary material for: The Complete Chloroplast Genome Sequence of Eupatorium fortunei: Genome Organization and Comparison with Related Species
Source: Genes (Basel). 2022 Dec 25;14(1):64. doi: 10.3390/genes14010064 (PMC9859021; doi:10.3390/genes14010064)
Supplement: Supplementary file 1 [file genes-14-00064-s001.zip › genes-2073348-supplementary.pdf]

Table S1. Chloroplast genome information table for 22 species in the article.

GenBank acc.	Species	Plastome size (bp)	Genus
OK545755.1	*Eupatorium fortunei*	152,401	*Eupatorium*
MT793847.1	*Ageratina fastigiata*	152,369	*Ageratina*
NC_015621.1	*Ageratina adenophora*	150,698	*Ageratina*
NC_031833.1	*Mikania micrantha*	152,092	*Mikania*
NC_023833.1	*Praxelis clematidea*	151,410	*Praxelis*
MT793836.1	*Mikania glomerata*	151,773	*Mikania*
MT793837.1	*Mikania haenkeana*	151,865	*Mikania*
MT793838.1	*Mikania lehmannii*	152,062	*Mikania*
MT793839.1	*Mikania neurocaula*	152,045	*Mikania*
MT793840.1	*Mikania oblongifolia*	151,845	*Mikania*
MT793841.1	*Mikania obtusata*	152,106	*Mikania*
MT793845.1	*Mikania triangularis*	151,956	*Mikania*
MT793846.1	*Stevia sp. Oliveira 769*	151,248	*Stevia*
MT793849.1	*Mikania additicia*	151,983	*Mikania*
MT793850.1	*Mikania brevifaucia*	152,161	*Mikania*
MT793851.1	*Mikania burchellii*	151,829	*Mikania*
MT793852.1	*Mikania fasciculata*	152,070	*Mikania*
MT793853.1	*Mikania purpurascens*	152,037	*Mikania*
MT793854.1	*Mikania smaragdina*	152,020	*Mikania*
MT793855.1	*Mikania sylvatica*	152,099	*Mikania*
NC_0356241	*Platycodon grandiflorus*	171,818	*Campanulaceae*
NC_026203.1	*Campanula takesimana*	169,551	*Campanulaceae*
